# Critical Point-of-care Ultrasound Diagnosis of Fournier’s Gangrene: A Case Report

**DOI:** 10.5811/cpcem.2021.11.54849

**Published:** 2022-01-28

**Authors:** Lauren Ramm, Kayla Guidry, Angela Cirilli, Ellen Kurkowski, Connie Yu

**Affiliations:** St. John’s Riverside Hospital, Department of Emergency Medicine, Yonkers, New York

**Keywords:** point-of-care ultrasound, Fournier’s gangrene, necrotizing infections, emergency medicine, case report

## Abstract

**Introduction:**

Fournier’s gangrene is a severe, necrotizing, and potentially fatal, soft tissue infection of the perineum that can be difficult to diagnose clinically. Point-of-care ultrasound (POCUS) has established a critical role in emergency medicine as a quick diagnostic tool due to its safety, accuracy, and cost effectiveness.

**Case Report:**

We present a case in which POCUS was used to rapidly confirm diagnosis in an unstable, severely septic patient presenting to the emergency department with Fournier’s gangrene.

**Conclusion:**

Point-of-care ultrasound can be used to make the diagnosis of Fournier’s gangrene in critical patients when other diagnostic modalities are not feasible due to a patient’s clinical state.

## INTRODUCTION

Fournier’s gangrene is a subset of necrotizing fasciitis most commonly caused by a polymicrobial infection of the perineal, genital, or perianal area. Affected patients are typically immunocompromised, with the most frequent comorbidities being diabetes mellitus and chronic alcohol abuse.^1^,^2^ Patients often present reporting only erythema and pain, making it tough to differentiate from scrotal cellulitis with physical exam alone.^2^ Visible necrosis and crepitus are typically late findings, and severe infection can develop rapidly despite the overlying skin appearance.^3^ There can be profound systemic findings out of proportion to the local extent of disease, eventually leading to shock, multiorgan system failure, and death.

Fournier’s gangrene is a synergistic necrotizing infection that produces leukocidal toxins and causes obliterating endarteritis that leads to micro-thrombophlebitis in the small subcutaneous vessels, thus leading to ischemia and necrosis as well as facilitating bacterial spread.^3^ Definitive treatment is immediate surgical debridement. Delay in treatment can result in significant morbidity, including extensive tissue loss, and mortality rates of 3–75%.^1^,^2^,^4^,^5^ Our case report shows the vital role point-of-care ultrasound (POCUS) played in early diagnosis and management of a hemodynamically unstable case of Fournier’s gangrene in the emergency department (ED).

## CASE REPORT

A 71-year-old male with past medical history significant for diabetes mellitus, coronary artery disease, chronic hypotension, hyperglycemia, chronic obstructive pulmonary disease, nicotine use, obstructive sleep apnea, and peripheral artery disease presented to the ED with right buttock and scrotal swelling for three days, associated with generalized weakness, shortness of breath, and urinary retention with dysuria. One day prior to presentation, the patient noticed non-traumatic bruising to his scrotum. He additionally reported increasing buttock pain that was not controlled by home oxycodone/acetaminophen 5/325 milligram (mg) tablets.

Upon arrival, his vital signs were notable for an oral temperature of 38.2° Celsius, blood pressure of 119/54 millimeters of mercury (mm Hg), and heart rate of 93 beats per minute. He was also tachypneic with a respiratory rate of 25 breaths per minute; oxygen saturation was 97% on room air. The physical examination was notable for an ill-appearing gentleman. He was awake and alert. Heart sounds were unremarkable aside from tachycardia, and lungs were clear to auscultation. Abdominal examination was soft, protuberant, and nontender. A focused genitourinary examination revealed an edematous, erythematous and exquisitely tender scrotum. Of note, there was a coin-sized, ecchymotic-appearing lesion on the scrotum with erythema and induration extending from the scrotum and perineum to the right buttock. No crepitus or fluctuance was palpated on examination.

Laboratory results were remarkable for leukocytosis to 15,000 thousand per millimeters cubed (K/mm3) (reference range: 4–10 K/mm^3^), lactic acidosis of 3.4 millimoles per liter (mmol/L) (0.4–2.0 mmol/L), marked acute kidney injury with creatinine of 5 mg per deciliter (mg/dL) (0.55–1.3mg/dL), and venous blood gas pH of 7.22 (7.310–7.410) secondary to the lactic acidosis and severe sepsis. The patient was mildly hyponatremic at 133 mmol/L (136–145 mmol/L) and hyperkalemic at 5.3 mmol/L (3.5–5.1 mmol/L), with the remainder of electrolytes within reference range. Intravenous antibiotics were initiated and emergent consultations with surgery and urology promptly obtained. Despite initial fluid resuscitation with administration of 30 milliliters per kilogram (ml/kg) of normal saline, the patient decompensated into septic shock. His blood pressure decreased to 88/41 mm Hg during the ED course; thus, he was not stable for transport or advanced imaging. By this time, the ecchymotic-appearing lesion and edema had expanded across his scrotum and perineum.

We used POCUS for the rapid assessment of the patient’s presumed clinical diagnosis of Fournier’s gangrene, a specific type of necrotizing fasciitis involving the perineum. Transverse and sagittal views of the perineum and scrotum were obtained using a high-frequency linear probe and revealed diffuse hyperechoic foci with posterior “dirty” shadowing, representative of subcutaneous air, and small fluid collections tracking along the fascial planes (Image).

**CPC-EM Capsule**

What do we already know about this clinical entity?

*Fournier’s gangrene is a severe, necrotizing, and potentially fatal, soft tissue infection of the perineum that can be difficult to diagnose clinically*.

What makes this presentation of disease reportable?

*We used point-of-care ultrasound (POCUS) to rapidly confirm Fournier’s gangrene in an unstable, severely septic patient in place of advanced imaging*.

What is the major learning point?

*Sonographic diagnosis of Fournier’s gangrene is a skill similar to other soft tissue diagnoses; POCUS can be performed looking for the signs identified in this case*.

How might this improve emergency medicine practice?

*Point-of-care ultrasound to diagnose Fournier’s gangrene and other necrotizing soft tissue infections has the potential to decrease the time to diagnosis and treatment*.

After confirming the diagnosis using POCUS imaging, the patient was taken directly from the ED to the operating room for immediate surgical debridement within three hours of his arrival at the hospital. An area of tissue measuring 25 centimeters (cm) × 30 cm that included both fascia and muscle was debrided from the scrotum, perineum, and right buttock. Dark, necrotic tissue and foul-smelling fluid were noted during surgery, consistent with Fournier’s gangrene. The patient was admitted to the intensive care unit until being transferred to a tertiary care center where he ultimately died of complications related to his illness.

## DISCUSSION

Fournier’s gangrene is an illness with significant morbidity and mortality. Diagnosis requires a high degree of clinical suspicion as it is often difficult to diagnose accurately based solely on examination. Physical examination often displays scrotal or perineal swelling, pain out of proportion to physical findings (72%), erythema (72%), edema beyond the area of erythema (75%), and crepitus (12–36%), but clinical diagnosis can be difficult in the early stages of the disease.^3^ Frequently, erythema and pain are the only initial presenting signs.^2^,^6^ Systemic inflammatory marker release can cause fever, tachycardia, and hypotension with potentially rapid progression to cardiovascular collapse and shock.^3^

If clinical suspicion is high for a necrotizing soft tissue infection, then early operative debridement is imperative as necrotic tissue and sepsis can progress rapidly.^1^ Current guidelines state the best initial radiographic examination is computed tomography (CT) with contrast, which can detect subcutaneous gas in soft tissues, a highly specific finding for necrotizing soft tissue infections, in addition to showing the source of infection.^7^ Due to rapid patient decline and hemodynamic instability, however, it may be difficult to obtain the necessary imaging to confirm the diagnosis without compromising patient safety. Additionally, intravenous contrast necessary to highlight surrounding inflammation, such as fat stranding, may not be possible due to significant kidney injury that frequently occurs with septic shock such as in this case.

Ultrasound is well known to be an excellent modality for detection of necrotizing soft tissue infections in other anatomical areas such as extremities.^5^ A few published articles have reported the diagnosis of Fournier’s gangrene using ultrasound, but all those cases were confirmed with subsequent CT or magnetic resonance imaging.^2^,^4^,^6^,^8^ Only one prior publication discussed the utility of POCUS performed by an emergency physician for rapid diagnosis of Fournier’s gangrene.^9^ Currently, the available literature describes POCUS as a useful tool to distinguish Fournier’s gangrene from other sonographically diagnosed causes of scrotal pain such as torsion, abscess, epididymitis, orchitis, testicular fracture, or incarcerated or strangulated hernia. Most authors suggest that confirmatory imaging is needed following sonographic evaluation of Fournier’s gangrene to show the extent of infection, presence of gas, and to guide surgical debridement.^4^,^6^,^8^

One prospective, observational study that looked at the accuracy of ultrasound in the diagnosis of necrotizing fasciitis demonstrated POCUS had a sensitivity of 88.2–100%, a specificity of 87.5–93.3%, a positive predictive value of 83.3%, and negative predictive value of 95.4% with an overall accuracy of 91.1%. Authors of the study reported that all the patients in the false negative group survived to discharge from the hospital, and all patients in the false positive group were ultimately diagnosed with cellulitis. The study was limited, however, to necrotizing infections of the limbs and excluded any patients who were suspected of having necrotizing infections of the scrotum.^5^,^7^ There is little, if any, data available to date regarding the sensitivity or specificity of POCUS for diagnosing necrotizing infections of the scrotum and perineum specifically.

Although testicular ultrasound for torsion is considered an advanced POCUS skill, diagnosis of Fournier’s gangrene is a skill that any emergency physician who has experience performing soft tissue ultrasound can and should be credentialed to perform. A high-frequency, linear transducer is used as is the case for most soft tissue examinations, using adequate gel. The scrotal tissue and surrounding inguinal tissue can then be surveyed looking for the classic findings of any necrotizing skin infection as seen in this case.

Sonographic findings include thickened subcutaneous tissues with characteristic hyperechoic foci with reverberation artifacts causing “dirty shadowing” indicative of subcutaneous gas. The underlying testes are spared as they have a separate blood supply. These findings are typically present before crepitus or air in the tissues can be appreciated clinically.^6^ In addition, ultrasound may display abnormal anechoic fluid collections between hyperechoic fascial planes, and this finding will often precede the presence of subcutaneous air, which is a late finding. All these findings can be remembered with the STAFF mnemonic, which stands for subcutaneous thickening, acoustic shadowing, and fascial fluid.^10^ There are few additional teaching tools regarding POCUS diagnosis of Fournier’s gangrene except for a few published case reports and radiologic resources; however, the diagnosis follows the same sonographic principles as taught and published for the diagnosis of necrotizing fasciitis in other areas of the body.^2^,^4^,^6^,^9^,^10^

Point-of-care ultrasound allows for rapid diagnosis of necrotizing soft tissue infections including Fournier’s gangrene and is readily available at the bedside, precluding the need for potentially dangerous transport of a critical patient out of the department. The time saved by making a diagnosis of Fournier’s gangrene with POCUS is significant compared to the time it takes to obtain advanced imaging such as CT with contrast, which can often be delayed in a busy ED for several hours due to various reasons. This time can be critical time saved to definitive surgical treatment and expedite the initiation of the appropriate resuscitation once this grave illness is recognized.

In this case, our patient presented with a relatively small area of visible necrosis and surrounding erythema and edema on physical exam but had a deeper infection. Performing POCUS allowed for rapid diagnosis of a deep infection and prompt surgical consult and debridement when CT imaging was not possible due to the patient’s hemodynamic instability and organ failure. This case highlights that using ultrasound routinely to confirm the diagnosis of Fournier’s gangrene and other necrotizing soft tissue infections has the potential to decrease the time to diagnosis and definitive treatment and to mobilize the surgical team, thereby improving patient outcomes. In addition, ultrasound imaging does not transmit radiation, is low cost, rapidly accessible, and allows the patient to safely remain in the ED.

## CONCLUSION

Clinical diagnosis of Fournier’s gangrene is often difficult and unreliable with physical examination and history alone, but it is a diagnosis that cannot be missed. The degree of disease is often hidden beneath the surface of benign-appearing cutaneous tissue until rapid hemodynamic decompensation occurs. Advanced imaging such as CT is currently the gold standard for diagnosis but can be impractical due to the instability of many patients affected, as in the case described here, in addition to added time, cost considerations, and exposure to radiation and contrast. This case highlights point-of-care ultrasound as a potential alternative and timesaving measure in critical patients. In addition, it may reduce the time to diagnosis and, therefore, mobilization of the appropriate specialist to decrease the overall time to treatment.

## Figures and Tables

**Image:**
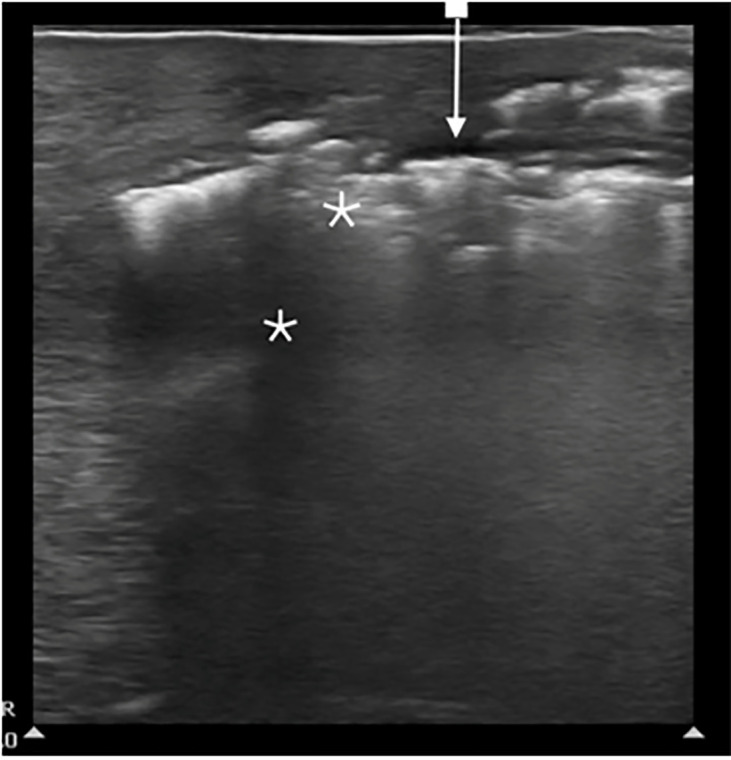
Ultrasound image using high-frequency linear probe showing presence of hypoechoic fluid collection (arrow) between fascial layers and underlying “dirty” hyperechoic A-lines with shadowing (*) indicating the presence of subcutaneous air.
